# The Hexosamine Biosynthetic Pathway as a Therapeutic Target after Cartilage Trauma: Modification of Chondrocyte Survival and Metabolism by Glucosamine Derivatives and PUGNAc in an Ex Vivo Model

**DOI:** 10.3390/ijms22147247

**Published:** 2021-07-06

**Authors:** Jana Riegger, Julia Baumert, Frank Zaucke, Rolf E. Brenner

**Affiliations:** 1 Division for Biochemistry of Joint and Connective Tissue Diseases, Department of Orthopedics, University of Ulm, 89081 Ulm, Germany; jana.riegger@uni-ulm.de (J.R.); julia.baumert@uni-ulm.de (J.B.); 2 Dr. Rolf M. Schwiete Research Unit for Osteoarthritis, Department of Orthopaedics (Friedrichsheim), University Hospital Frankfurt, Goethe University, 60528 Frankfurt/Main, Germany; Frank.Zaucke@kgu.de

**Keywords:** hexosamine biosynthetic pathway, cartilage trauma, post-traumatic osteoarthritis, chondrocytes, O-GlcNAcylation, glucosamine, cell death, therapy

## Abstract

The hexosamine biosynthetic pathway (HBP) is essential for the production of uridine diphosphate N-acetylglucosamine (UDP-GlcNAc), the building block of glycosaminoglycans, thus playing a crucial role in cartilage anabolism. Although O-GlcNAcylation represents a protective regulatory mechanism in cellular processes, it has been associated with degenerative diseases, including osteoarthritis (OA). The present study focuses on HBP-related processes as potential therapeutic targets after cartilage trauma. Human cartilage explants were traumatized and treated with GlcNAc or glucosamine sulfate (GS); PUGNAc, an inhibitor of O-GlcNAcase; or azaserine (AZA), an inhibitor of GFAT-1. After 7 days, cell viability and gene expression analysis of anabolic and catabolic markers, as well as HBP-related enzymes, were performed. Moreover, expression of catabolic enzymes and type II collagen (COL2) biosynthesis were determined. Proteoglycan content was assessed after 14 days. Cartilage trauma led to a dysbalanced expression of different HBP-related enzymes, comparable to the situation in highly degenerated tissue. While GlcNAc and PUGNAc resulted in significant cell protection after trauma, only PUGNAc increased COL2 biosynthesis. Moreover, PUGNAc and both glucosamine derivatives had anti-catabolic effects. In contrast, AZA increased catabolic processes. Overall, “fueling” the HBP by means of glucosamine derivatives or inhibition of deglycosylation turned out as cells and chondroprotectives after cartilage trauma.

## 1. Introduction

As the most common joint disease in the elderly population and one of the leading causes of disability in age, osteoarthritis (OA) has a high impact on today’s society [1]. Preceding injuries are considered a major risk factor in joint degeneration, causing a special form called post-traumatic OA (PTOA), which can also affect the younger population and accounts for about 10% of the total incidence of knee OA [2].

The pathogenesis of PTOA is induced by a traumatic impact causing a sudden increase of cell death and subsequent synovial inflammation. The surviving chondrocytes secrete excessive amounts of catabolic enzymes, such as matrix metalloproteinases (MMPs) and proteases of the ADAMTS (a disintegrin and metalloproteinase with thrombospondin motifs) family, which contribute to the breakdown of the main cartilage components type II collagen and aggrecan [3]. These catabolic processes can persist over years, driving progressive cartilage degeneration. 

Despite huge scientific efforts, the development of efficient drugs, preventing or delaying the onset of PTOA, remains difficult, in particular due to the complex pathomechanisms involved. Most therapeutic approaches, such as non-steroidal anti-inflammatory drugs (NSAIDs), focus on symptomatic relief and represent just a temporary solution [4]. Besides NSAIDs, which can also be considered rapid-acting symptom modifying osteoarthritis drugs (SMOADS), there are slow-acting drugs, also referred to as symptomatic slow acting drugs for osteoarthritis (SYSADOA), including cytokine modulators, such as diacerein, glucosamine, and chondroitin as precursors of matrix components, as well as hyaluronan (also known as hyaluronic acid) [5,6].

**Figure 1:**
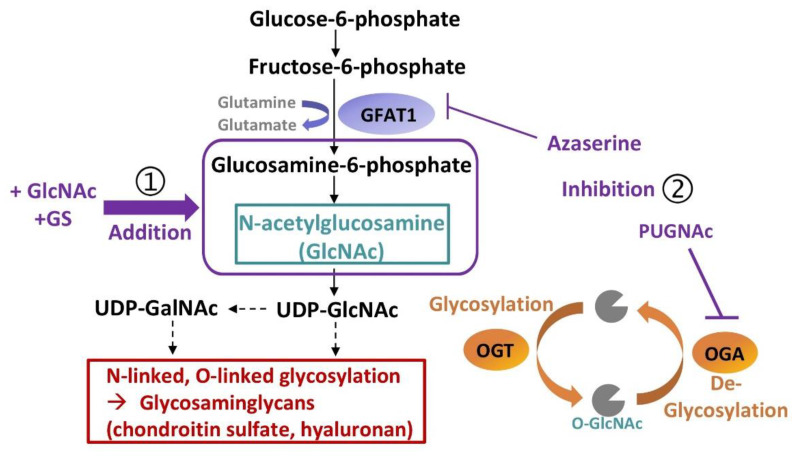
Schematic illustration of the hexosamine biosynthetic pathway (HBP), its role in glycosaminoglycan synthesis and potential therapeutic targeting. In the present study, the HBP is targeted in different ways: (1) glucosamine derivatives (N-acetylglucosamine (GlcNAc) or glucosamine sulfate (GS)) were added at different concentrations to enhance the bioavailability of the substrate for uridine diphosphate GlcNAc (UDP-GlcNAc) generation; (2) the rate-limiting enzyme, glutamine fructose-6-phosphate amidotransferase (GFAT1), was inhibited by means of Azaserine, while hydrolysis of O-GlcNAc residues (de-glycosylation) was suppressed using PUGNAc, a specific inhibitor of OGA. GalNAc = N-Acetylgalactosamine, OGA = N-acetylglucosaminidase, OGT = O-GlcNAc transferase, PUGNAc = O-(2-acetamido-2deoxy-D-glucopyranosylidene)amino-N-phenylcarbamate.

These matrix precursors and hyaluronan have one thing in common; they contribute to or are derived from the hexosamine biosynthetic pathway (HBP), which plays a substantial role in cartilage homeostasis [7]. In principle, the HBP is a branch of glycolysis, although it is not involved in energy generation but production of uridine diphosphate N-acetylglucosamine (UDP-GlcNAc). The rate-limiting enzyme, glutamine fructose-6-phosphate amidotransferase (GFAT-1), converts fructose-6-phosphate into glucosamine 6-phosphate, the precursor of GlcNAc (Figure 1). GlcNAc is the essential building block for the biosynthesis of glycosaminoglycans (GAGs), such as keratan sulfate, chondroitin sulfate, and hyaluronan, which represent about 90% of the total mass of aggrecan, the most abundant proteoglycan (PG) in articular cartilage [8]. The transfer of GlcNAc to proteins represents a special form of glycosylation (O-GlcNAcylation) and requires energy, enabled by coupling the monosaccharide with the nucleotide UDP. O-GlcNAcylation is not only essential for PG synthesis but also functions as a regulatory mechanism for various cellular processes [9,10]. This post-transcriptional modification is controlled by two enzymes: O-GlcNAc transferase (OGT), which drives the addition of UDP-GlcNAc to proteins, and the N-acetylglucosaminidase (OGA or O-GlcNAcase), which reverses the reaction. O-GlcNAcylation is highly responsive towards different stimuli, including trauma and cytokines [11], and has been found to be dysregulated in OA and other age-related degenerative diseases [11,12,13].

Concerning the fundamental importance of the HBP for the biosynthesis of matrix components, it might be rational to increase the substrate availability via glucosamine supplementation as a therapeutic approach in OA. In fact, there are various promising in vitro studies reporting anabolic, antioxidative, anti-catabolic (chondroprotective), pro-mitotic, and anti-inflammatory effects of glucosamines on chondrocytes [14,15,16,17]. However, the overall efficacy of glucosamines in OA therapy remains controversially discussed due to contradictory results, especially with regard to clinical trials [4,18,19,20].

Despite the central role of the HBP and protein O-GlcNAcylation in cartilage, the influence of a traumatic single impact as a potential trigger or suppressor of HBP-related processes in cartilage tissue has not been taken into account so far. Therefore, this study will not directly focus on potential therapeutic effects of the glucosamine derivatives GlcNAc and glucosamine sulfate (GS) but rather considers the HBP as a whole to investigate its possible involvement in post-traumatic processes and its relevance as a therapeutic target after cartilage trauma in particular.

## 2. Results

### 2.1. Gene Expression of HBP-Related Enzymes Is Altered after Cartilage Trauma and in Highly Degenerated Tissue

**Figure 2:**
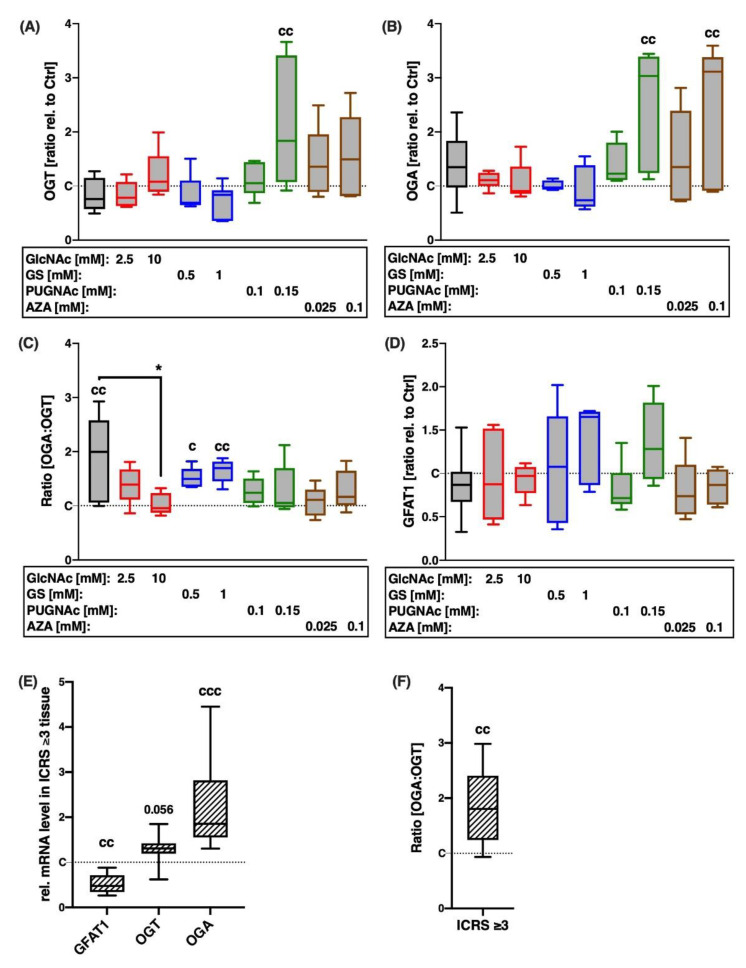
Gene expression levels of HBP-related enzymes 7 days after trauma and in highly degenerated tissue. Gene expression of HBP-related enzymes was evaluated in (**A**,**B**,**D**), impacted and subsequently treated cartilage explants, as well as (**E**) highly degenerated cartilage tissue (ICRS grade ≥ 3). Moreover, the ratio of OGA to OGT was calculated for (**C**) impacted and subsequent treated cartilage explants, as well as (**F**) OA cartilage. Statistical analysis: (**A**,**B**,**D**) one-way ANOVA, (**C**) the Kruskal–Wallis test, (**E**) multiple *t*-tests, (**F**) unpaired two-tailed *t*-test. Significant differences between groups were depicted as: [versus T] * = *p* < 0.05; [versus C] c = *p* < 0.05, cc = *p* < 0.01; all data sets *n* ≥ 5; ICRS grade ≥ 3 tissue *n* ≥ 6. Shaded boxes = traumatized cartilage explants (T), striped boxes = highly degenerated cartilage tissue (ICRS grade ≥ 3).

Human cartilage tissue explants were subjected to a single impact load of 0.59 J using a drop-tower model, as previously described [21]. This trauma suppressed gene expression levels of OGT by 25%, while increasing that of OGA by 35% (Figure 2A,B), thus significantly changing the ratio of OGA to OGT ([vs. C] 2-fold; Figure 2C). This alteration was markedly reduced by GlcNAc, PUGNAc, and AZA; however, the attenuating effect was only significant in the case of 10 mM GlcNAc. Despite increased gene expression of OGA in presence of its inhibitor PUGNAc, mRNA levels of OGT were likewise enhanced, resulting in an overall alleviated ratio of OGA to OGT, which was comparable to the control level. While trauma and subsequent treatment had no significant effect on mRNA levels of GFAT-1 in macroscopically intact cartilage (Figure 2D), gene expression analysis of highly degenerated cartilage tissue (ICRS score ≥ 3) revealed a significant reduction in GFAT-1 expression of about 50% compared to macroscopically intact tissue (ICRS score ≤ 1) (Figure 2E). Moreover, the ICRS score ≥ 3 tissue exhibited an about 1.8-fold increased ratio of OGA to OGT (Figure 2F).

### 2.2. GlcNAc and PUGNAc Exert Cell Protective Effects after Cartilage Trauma

**Figure 3:**
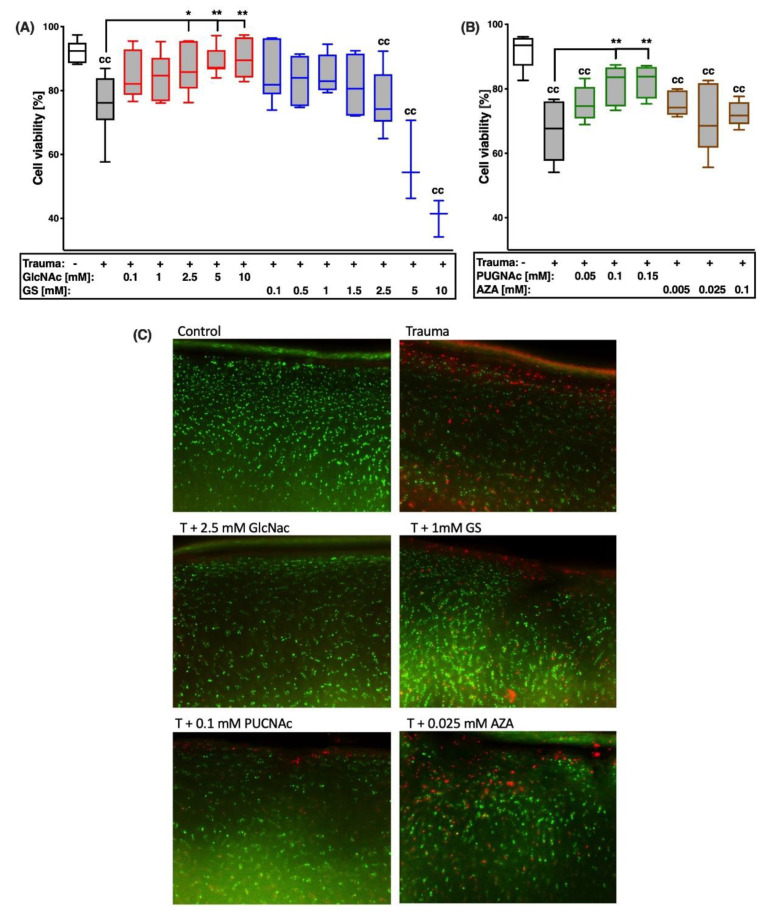
Effects of glucosamine treatment or addition of inhibitors on cell viability 7 days after cartilage trauma. Impacted cartilage explants were treated with different concentrations of (**A**) glucosamine derivatives GlcNAc or GS or (**B**) OGA-inhibitor PUGNAc and GFAT-1-Inhibitor AZA, respectively, for 7 days. (**C**) Exemplary fluorescence images of live/dead staining. Statistical analysis: (**A**,**B**) one-way ANOVA. Significant differences between groups were depicted as: [versus T] * = *p* < 0.05, ** = *p* < 0.01; [versus C] cc = *p* < 0.01; all data sets *n* ≥ 5, except for 5 mM and 10 mM GS, *n* = 3. Blank box = unimpacted control (C), shaded boxes = traumatized cartilage explants (T).

To investigate potential involvement of the HBP in the regulation of cell death and survival after cartilage trauma, cell viability was assessed by means of a live/dead staining (Figure 3C). 7 days after trauma, the cell viability was significantly decreased by about 20% ([vs. C] Figure 3A: −16.8%, Figure 3B: −25%). While treatment with GlcNAc revealed significant cell protective effects ([vs. T] 2.5 mM: +11.3%; 5 mM: +13.6%; 10 mM: +14.6%), no improvement, except further reduction of the cell viability, was observed in the case of high-dose GS administration (Figure 3A). Due to the cell toxic effects observed for high concentrations (5 and 10 mM) of GS, these conditions were only exemplarily tested (*n* = 3).

While the addition of GFAT-inhibitor AZA had no significant effect on the percentage of living cells after cartilage trauma, inhibition of OGA by PUGNAc significantly promoted the survival of chondrocytes ([vs. T] 0.1 mM: +14.4%; 0.15 mM: +15.7%; Figure 3B).

### 2.3. While PUGNAc Revealed Chondroanabolic Effects, Glucosamine Derivatives GlcNAc and GS Suppressed Type II Collagen Synthesis after Cartilage Trauma

**Figure 4:**
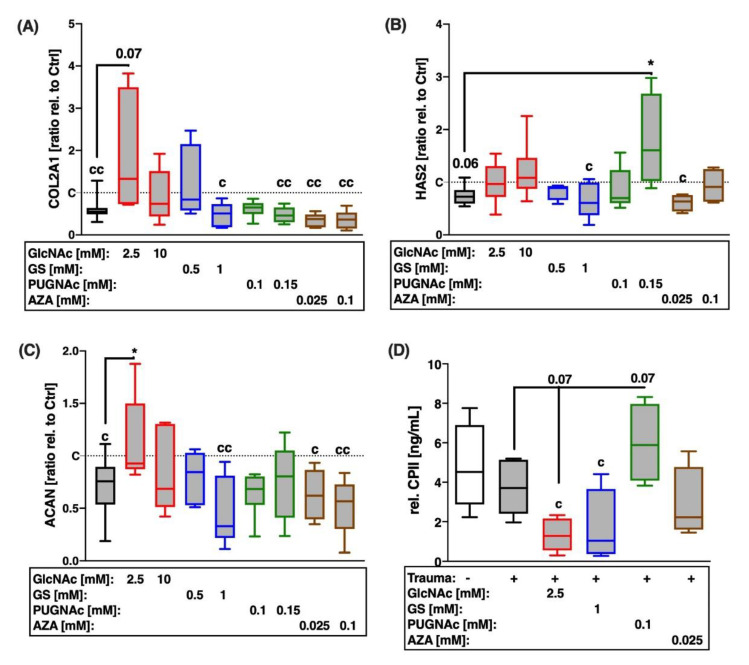
Effects of glucosamine, PUGNAc, and AZA on chondroanabolic processes 7 days after cartilage trauma. Chondroanabolism was evaluated by means of gene expression analysis of (**A**) COL2A1, (**B**) HAS2, and (**C**) ACAN, as well as by (**D**) quantification of CPII release. Statistical analysis: (**A**,**B**) Kruskal–Wallis test, (**C**,**D**) one-way ANOVA. Significant differences between groups were depicted as: [versus T] * = *p* < 0.05; [versus C] c = *p* < 0.05, cc = *p* < 0.01; all data sets *n* ≥ 5. Blank box = unimpacted control (C), shaded boxes = traumatized cartilage explants (T).

Possible involvement of the HBP in chondroanabolic effects after cartilage trauma was addressed by gene expression analysis of COL2A1, hyaluronan synthase 2 (HAS2), and aggrecan, as well as quantification of CPII, which reflects the actual biosynthesis of type II collagen (Figure 4).

Compared to the unimpacted cartilage, chondroanabolic gene expression was significantly reduced after trauma (Figure 4A–C). Although, treatment with 2.5 mM GlcNAc enhanced the gene expression of COL2A1 ([vs. T] 2.4-fold) and ACAN ([vs. T] 1.6-fold), both glucosamine derivatives suppressed the biosynthesis of type II collagen ([vs. C] GlcNAc: −3.5 ng/mL; GS: −3 ng/mL; Figure 4D). Moreover, 1 mM GS further reduced gene expression of ACAN.

While AZA had rather anti-anabolic effects, as demonstrated by reduced biosynthesis of type II collagen ([vs. C] −2.3 ng/mL), PUGNAc significantly induced both gene expression of HAS2 ([vs. T] 2.4-fold) and the release of CPII ([vs. T] +2.2 ng/mL), despite unchanged mRNA levels of COL2A1.

### 2.4. Trauma-Induced Expression of MMPs and Subsequent Type II Collagen Degradation Are Markedly Decreased after Treatment with Glucosamines or PUGNAc

**Figure 5:**
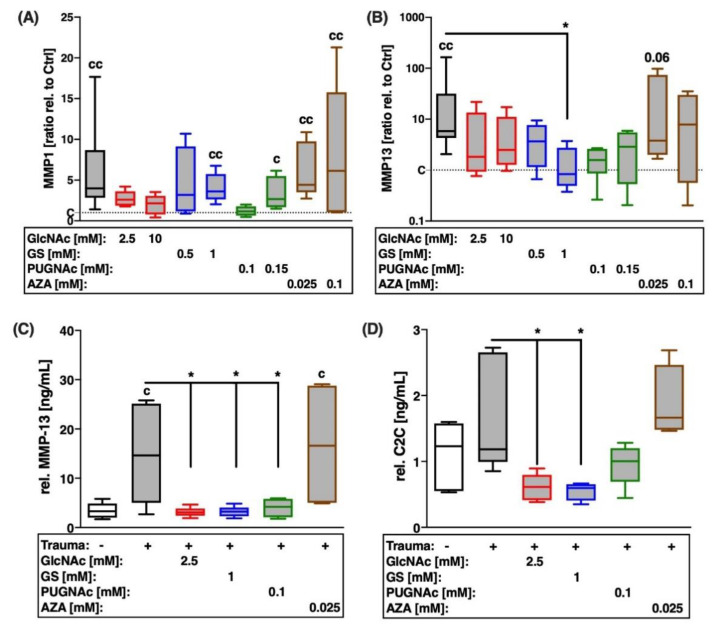
Effects of glucosamine treatment or addition of inhibitors on trauma-induced expression of collagenases and COL2A breakdown 7 days after trauma. Catabolic processes were assessed by the means of gene expression analysis of (**A**) MMP-1 and (**B**) MMP-13, as well as (**C**) quantification of MMP-13 release and (**D**) type II collagen breakdown product C2C. Statistical analysis: (**A**,**B**) Kruskal–Wallis test, (**C**,**D**) one-way ANOVA. Significant differences between groups were depicted as: [versus T] * = *p* < 0.05; [versus C] c = *p* < 0.05, cc = *p* < 0.01; all data sets *n* ≥ 5. Blank box = unimpacted control (C), shaded boxes = traumatized cartilage explants (T).

**Figure 6:**
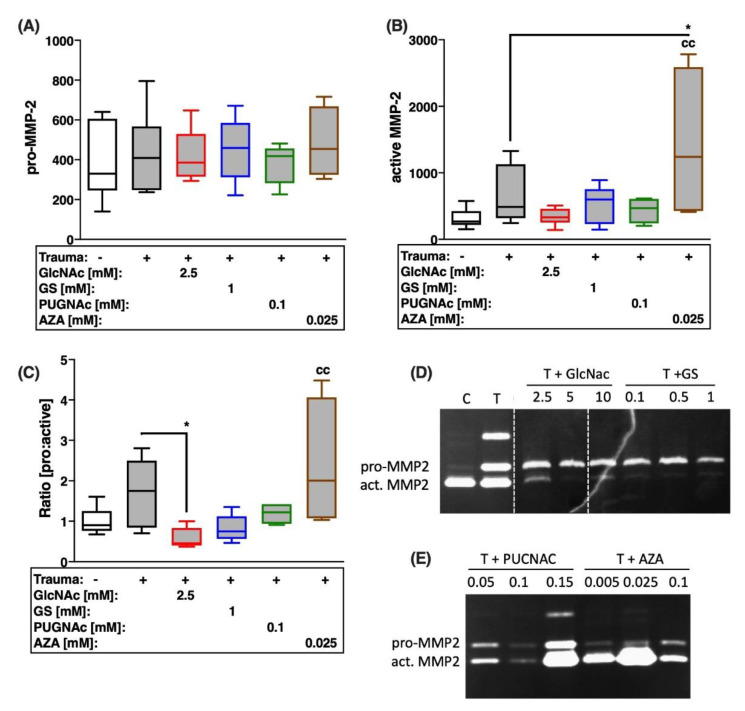
Effects of glucosamine treatment or addition of inhibitors on trauma-induced secretion and activation of MMP-2 7 days after trauma. Release of (**A**) pro-MMP-2 and (**B**) active MMP-2 was measured by means of gelatin zymography, as exemplarily demonstrated in (**D**,**E**). (**C**) Ratio of zymographically detectable pro-MMP-2 to active MMP-2. Statistical analysis: (**A**–**C**) one-way ANOVA. Significant differences between groups were depicted as: [versus T] * = *p* < 0.05; [versus C] cc = *p* < 0.01; all data sets *n* ≥ 5. Blank box = unimpacted control (C), shaded boxes = traumatized cartilage explants (T).

Possible influence of the HBP on trauma-induced expression of MMPs and subsequent breakdown of type II collagen was evaluated by gene expression analysis of MMP-1 and -13 (Figure 5A,B), quantification of secreted MMP-2 (zymographical detection; Figure 6 and MMP-13 (Figure 5C), as well as the degradation product of type II collagen (C2C), generated by MMP-1, -8, and -13 (Figure 5D).

Cartilage trauma resulted in excessive gene expression of ECM-degrading MMPs ([vs. C] MMP1: 4-fold; MMP13: 5.8-fold), though concentration of C2C was not significantly enhanced compared to the unimpacted control. Both glucosamine derivatives exhibited anti-catabolic effects to varying degrees. While GlcNAc had stronger suppressive effects, with respect to the gene expression of MMP-1 (Figure 5A), GS revealed higher efficacy in the case of MMP-13 (Figure 5B). However, GlcNAc and GS demonstrated equal anti-catabolic and chondroprotective effects, respectively, concerning the secretion of MMP-13 ([vs. T] GlcNAc: −11.54 ng/mL; GS: −11.8 ng/mL; Figure 5C) and subsequent degradation of type II collagen ([vs. T] both −0.97 ng/mL; Figure 5D). Moreover, GlcNAc significantly reduced the conversion of pro-MMP-2 to active MMP-2 by 2.9-fold (Figure 6C).

Although, comparable anti-catabolic effects were found after inhibition of OGA by means of PUGNAc, as demonstrated by decreased secretion of MMP-13 ([vs. T] −11 ng/mL), the reduction of the degradation product C2C was less pronounced ([vs. T] −0.57 ng/mL). While no significant changes could be observed for the secretion of pro-MMP2 (Figure 6A), the addition of AZA increased the zymographically detectable amount of active MMP-2 ([vs. C] 4-fold; [vs. T] 3-fold; Figure 6B) and the generation of C2C ([vs. T] +0.5 ng/mL) after cartilage trauma.

### 2.5. Trauma-Induced Expression of Aggrecanases and Subsequent Aggrecan Degradation Are Largely Decreased after Treatment with Glucosamines or PUGNAc

Influence of the HBP on trauma-induced expression and proteolytic activity of aggrecanases was determined by the gene expression analysis of ADAMTS4 and 5, estimation of the aggrecanase activity, and the histological assessment of PG by means of Safranin-O staining.

**Figure 7:**
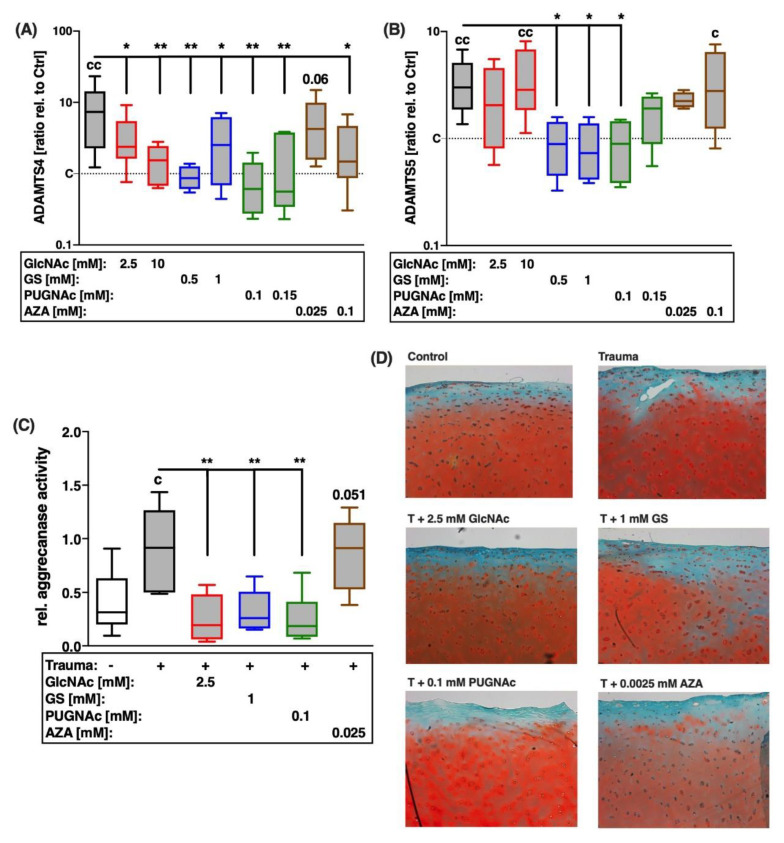
Effects of glucosamine treatment or addition of inhibitors on trauma-induced expression of aggrecanases and PG breakdown 7 days and 14 days after trauma. Catabolic processes were assessed by means of gene expression analysis of (**A**) ADAMTS-4 and (**B**) ADAMTS-5, as well as (**C**) quantification of relative aggrecanase activity (7 days after trauma). Moreover, (**D**) PG content in cartilage explants 14 days after trauma and corresponding treatments were evaluated by means of exemplary images of cartilage after Saf-O staining. Statistical analysis: (**A**–**C**) one-way ANOVA. Significant differences between groups were depicted as: [versus T] * = *p* < 0.05, ** = *p* < 0.01; [versus C] c = *p* < 0.05, cc = *p* < 0.01; all data sets *n* ≥ 5. Blank box = unimpacted control (C), shaded boxes = traumatized cartilage explants (T).

After cartilage trauma, gene expression levels of ADAMTS-4 and -5 were significantly enhanced ([vs. C] ADAMTS4: 7.4-fold; ADAMTS5: 3-fold; Figure 7A,B). This was reflected in increased aggrecanase activity ([vs. C] 2.9-fold; Figure 7C), which can be considered proportional to the total aggrecanase amount. Additionally, histological assessment of PG by means of Saf-O staining confirmed the increased loss of PG in traumatized cartilage explants (Figure 7D). Treatment with glucosamines or PUGNAc significantly suppressed trauma-induced gene expression of aggrecanases, as well as aggrecanase activity ([vs. T] GlcNAc: −4.8-fold; GS: −3.5-fold; PUGNAc: −4.9-fold). These anti-catabolic effects and the subsequent preservation of PG could be confirmed in the corresponding Saf-O staining of GlcNAc and PUGNAc treated cartilage 14 days after trauma. In contrast, AZA had no additional effect on the expression levels of aggrecanases after trauma, though resulted in strong PG depletion, as demonstrated in the severe de-staining of the cartilage matrix.

## 3. Discussion

The HBP is essentially responsible for the generation of UDP-GlcNAc, the building block for posttranscriptional O-GlcNAcylation, which is not only involved in the biosynthesis of PG but also in the regulation of various metabolic processes [9,10]. Despite its crucial role in cartilage homeostasis, the actual implication of the HBP during OA development and, in particular, after traumatic cartilage injury remains largely unknown. 

In the present study, we investigated the impact of targeted manipulation of the HBP in various ways. First, we added two different glucosamine derivatives, GS and GlcNAc, in order to increase the substrate availability and potentially boost HBP activity. Second, we inhibited the enzymatic activity of OGA via PUGNAc, thus impairing reversibility of O-GlcNAcylation and causing accumulation of O-glycosylated proteins. Third, we directly inhibited the rate-limiting enzyme of the HBP, GFAT-1, by means of AZA in order to suppress the generation of UDP-GlcNAc and therefore reducing subsequent O-GlcNAcylation. Unlike previous studies, which mainly focus on glucosamine administration as a therapeutic approach against advanced and symptomatic OA, this is the first study evaluating targeted modulation of the HBP after a singular cartilage impact using a human ex vivo trauma model. The experimental design allows investigation of HBP-related processes as a possible therapeutic target after mechanical injury and subsequent cartilage degeneration on cellular and molecular level, which in vivo might promote the development of a PTOA.

Some years ago, glucosamines, and in particular GS, have been propagated to be an ideal therapeutic approach in OA treatment, combining both pain relief and cartilage regeneration. Accordingly, glucosamine application was highly recommended in the guidelines of the European League Against Rheumatism (EULAR) for the management of knee OA in 2003 [21]. In fact, different reviews and meta-analyses between 2003 and 2008 summarized the outcomes of various clinical trials on GS and concluded that it was effective in pain reduction, improvement of the joint functionality, as well as the overall reduction of OA progression and subsequent risk of joint replacement [22]. About 10 years later, the Osteoarthritis Research Society (OARSI) guidelines stated the recommendation about glucosamine treatment as “uncertain” in case of symptom relief and “not appropriate” for disease modification [4]. Taken together, therapeutic efficacy of glucosamines in OA disease remains controversial, especially because of the inconsistency between study reports deriving from industry-sponsored and independent trials, as well as a generally large heterogeneity among studies [4]. Nevertheless, as mentioned above, we investigated a novel aspect in glucosamine administration and therapeutic targeting of the HBP, respectively, with a focus on attenuation of trauma-induced pathomechanisms and thus prevention of PTOA development. In our study, GlcNAc exhibited cell protective effects after cartilage trauma; however, this could not be confirmed in the case of GS, which led to enhanced cell death in doses higher than 1.5 mM. In fact, both cell protective and as cytotoxic effects of glucosamines have been previously reported. On the one hand, glucosamine has been found to induce autophagy in chondrocytes, which is considered to facilitate cell survival and protect against cartilage degeneration [20,23,24,25]. On the other hand, long-term exposure and high glucosamine concentrations were found to promote mitochondrial and peroxisomal dysfunction, as well as accumulation of very long chain fatty acids (VLCFA) [20]. Therefore, glucosamines might act as inducers and suppressors of autophagic and apoptotic processes at the same time, possibly depending on the respective experimental conditions. Interestingly, cytotoxic effects have been reported for glucosamines (i.e., glucosamine hydrochloride (GlcN-HCl)), [20,26,27] but not in case of GlcNAc. In contrast to GlcNAc, glucosamine has been found to be actively internalized by chondrocytes via glucose transporters (GLUTs), thus inhibiting glucose uptake and leading to depleted intracellular ATP stores [28]. Shikhman et al. supposed that GlcNAc interacts with GLUTs, although positively affecting their affinity for glucose, the main source for energy supply and precursor of GAG synthesis in chondrocytes [28]. 

In line with Uitterlinden et al., who reported both anti-catabolic and anti-anabolic effects of GS and GlcN-HCl in OA cartilage [16], we found significant chondroprotective effects for GlcNAc and GS after cartilage trauma, but also observed an unexpected decline in CPII. On first sight, this finding contradicts the general opinion that glucosamine enhances the ECM synthesis in cartilage; however, post-translational modification of type II collagen does not depend upon the HBP or UDP-GlcNAc, respectively [29]. Therefore, the suppressive effects of glucosamines clearly deserve further investigation. While GS suppressed the gene expression of ACAN and HAS2, GlcNAc increased the corresponding mRNA levels to some extent, depending on the concentration. In accordance with this, GlcNAc has previously been found to accelerate the expression of HAS2 and subsequent synthesis of hyaluronan, while glucosamine resulted in opposite effects [16,28]. The differential impact of GlcNAc and GS on chondroanabolism might also account for divergent findings concerning the PG content, as demonstrated by Saf-O staining. Despite similar suppression of aggrecanases and MMPs found for both glucosamine derivatives, higher PG decline was found in GS treated cartilage explants compared to GlcNAc treated explants.

Anti-catabolic effects of glucosamines have been previously reported, though mainly addressing interleukin 1b (IL-1b)-mediated MMP and ADAMTS expression [14,30,31]. Nevertheless, similar to cytokine-induced catabolism, the therapeutic effects of glucosamine administration after cartilage trauma might result from the inhibition of OA-associated mitogen-activated protein kinase (MAPK) pathways, including c-Jun N-terminal kinase and p38, as shown for GS [32]. However, inhibition of IL-1b-induced catabolism by GlcNAc has not been found to have any effect on ERK, JNK, or p38 MAPK pathways [33]. Moreover, spontaneous (i.e., age-dependent) and induced (i.e., via OGA-inhibitor thiamet-G) accumulation of O-GlcNAcylated proteins has even been associated with enhanced MAPK phosphorylation [34,35]. This activation correspondingly induced both MMP expression and chondrogenic differentiation of ATDC5 cells at the same time [34]. These contradictory observations imply a dual effect of O-GlcNAcylation on MAPK signaling, which clearly deserves further investigation with regard to cartilage trauma and OA development.

In our study, PUGNAc administration resulted not only in cell and chondroprotective effects but also in chondroanabolic effects after cartilage trauma, implying a positive effect by OGA-inhibition and subsequent accumulation of O-glycosylated proteins. In line with this, inhibition of GFAT-1 by means of AZA was found to result in opposite effects. Hence, we concluded that HBP functionality and O-GlcNAcylation, respectively, is essential for cartilage homeostasis and provides protection against trauma-induced pathomechanisms. Accordingly, expression of OGT and O-GlcNAcylation is thought to be responsive to cellular stress, comprising oxidative stress, ER stress, ischemia reperfusion injury, and more; however, only little is known about regulation of OGA in injured cells [36,37]. Stress-induced upregulation of OGT leads to enhanced O-GlcNAc levels and can be considered a pro-survival signaling program, while, in contrast, reduced O-GlcNAcylation increases susceptibility of cells and tissues to injury [36,38,39]. Surprisingly, cartilage trauma did not induce the gene expression of OGT but OGA, which was also observed in highly degenerated tissue (ICRS score ≥ 3). Comparable findings were described by Tardio et al., who determined a similar alteration of the OGA to OGT ratio in cartilage of OA patients, as well as after IL-1b stimulation of isolated OA chondrocytes [13]. It might be possible that the responsiveness of this pro-survival signaling program decreases with age. In fact, aging has a high impact on protein O-GlcNAcylation and vice-versa. Accumulation of O-GlcNAc modified proteins has been associated with development and progression of various age-related diseases and was found in OA cartilage tissue, despite enhanced OGA levels [12,13]. Comparable accumulation of O-GlcNAc modified proteins has also been described in aged retina; however, though, the addition of PUGNAc or UDP-GlcNAc and subsequent enhancement of O-GlcNAcylation reduced intracellular oxidative stress levels [35], which corroborates our findings.

Direct inhibition of GFAT-1 by AZA, and thus attenuation of all HBP-associated processes, resulted in enhanced expression of catabolic enzymes and subsequent ECM destruction after cartilage trauma. This was demonstrated by increased concentrations of type II collagen breakdown product C2C and severe PG depletion in Saf-O stained cartilage explants. However, loss of PG in AZA-treated cartilage explants might also be a result of both enhancement of protease activity and decrease of ECM synthesis, as shown in reduced gene expression of COL2A1, HAS2, and ACAN, as well as type II collagen synthesis. There is only little known about the impact of GFAT-1 inhibition by AZA or any other component in cartilage; however, Honda et al. reported significant impairment in the synthesis of cartilage-characteristic PG-H in chicken embryos exposed to AZA [40].

## 4. Materials and Methods

### 4.1. Specimen Preparation and Cultivation Conditions

Overall, macroscopically intact tissue samples (International Cartilage Repair Society (ICRS) score ≤ 1) from femoral condyles of 15 patients (mean age 63 years; 8 male and 7 female patients) were included in the study. Human cartilage was obtained from donors undergoing total knee joint replacement due to OA. Informed consent was obtained from all patients according to the terms of the Ethics Committee of the University of Ulm. Full-thickness cartilage explants (Ø = 6 mm) were harvested, weighed, and cultivated in serum-containing medium (1:1 DMEM/Ham’s F12 supplemented with 10% fetal bovine serum, 0.5% penicillin/streptomycin (PAA Laboratories, Pasching, Austria), 0.5% L-glutamine, and 10 μg/mL 2-phospho-L-ascorbic acid trisodium salt) for 24 h in an incubator (37 °C, 5% CO_2_, 95% humidity). Afterwards, the explants were traumatized and cultivated for 7 days and 14 days (only for histological assessment), respectively, in serum-free medium (DMEM supplemented with 1% sodium pyruvate, 0.5% L-glutamine, 1% non-essential amino acids, 0.5% penicillin/streptomycin, 10 μg/mL 2-phospho-L-ascorbic acid trisodium salt, and 0.1% insulin-transferrin-sodium selenite (Sigma-Aldrich, Taufkirchen, Germany)). All chemicals were purchased from Biochrom (Berlin, Germany), unless specified otherwise. Additionally, highly degenerated (ICRS score ≥ 3) and corresponding macroscopically intact tissue samples (ICRS grade ≤ 1) of 7 patients (mean age 67 years) were immediately snap frozen for RNA isolation to evaluate degeneration-associated changes in gene expression. 

### 4.2. Impact Loading and Subsequent Treatment

The cartilage explants were subjected to a single impact load of 0.59 J using a drop-tower model, as previously described [41], and further cultivated under serum-free conditions (described above). Unloaded explants served as controls. Impacted cartilage explants were treated with the following additives (Sigma-Aldrich) for 7 and 14 days, respectively: GlcNAc or GS (each 0.1–10 mM), an inhibitor of O-GlcNAcase (PUGNAc (O-(2-acetamido-2deoxy-D-glucopyranosylidene)amino-N-phenylcarbamate): 0.05–0.15 mM) or the GFAT1-inhibitor azaserine (AZA: 0.005–0.1 mM) (Figure 1). Fresh additives were added concomitantly with medium change every 2–3 days.

### 4.3. Live/Dead Cell Cytotoxicity Assay

To determine the percentage of viable cells 7 days after trauma, a live/dead viability/cytotoxicity assay (Molecular Probes, Invitrogen) was performed, as previously described [42]. In short, unfixed tissue sections (0.5 mm thickness) were stained with 1 µM calcein AM and 2 µM ethidium homodimer-1 for 30 min. After washing in PBS, they were microscopically analyzed by means of a z-stack module (software AxioVision, Carl Zeiss, Jena, Germany). Three pictures were made from each tissue section. All cells on the picture were counted manually (Image J software version 1.42q). The average count per picture was about 2000 cells.

### 4.4. mRNA Isolation and cDNA Synthesis

For total RNA isolation 7 days after trauma, cryopreserved cartilage explants were pulverized with a microdismembrator S (B. Braun Biotech, Melsungen, Germany). Subsequently, RNA was isolated using the Lipid Tissue Mini Kit (Qiagen, Hilden, Germany). RNA was reverse transcribed with the Omniscript RT Kit (Qiagen) and used for quantitative real-time PCR analysis (StepOne-PlusTM Real-Time PCR System, Applied Biosystems, Darmstadt, Germany).

### 4.5. Quantitative Real-Time Polymerase Chain Reaction (qRT-PCR)

Determination of the relative expression levels was performed by means of qRT-PCR analysis using the ∆∆ Ct method. To detect desired sequences, a TaqManTM Gene Expression Master Mix for TaqMan Gene Expression Assay (both Applied Biosystems) was used for the following probes: Hs00192708_m1 (ADAMTS4), Hs00199841_m1 (ADAMTS5), Hs00264051_m1 (COL2A1), Hs00899865_m1 (GFAT-1), Hs00193435_m1 (HAS2), Hs02800695_m1 (HPRT1), Hs00899658 (MMP-1), Hs00233992_m1 (MMP-13), Hs01028844_m1 (OGA/MGEA5), and Hs00269228_m1 (OGT). Power SYBR Green PCR Master Mix (Applied Biosystems) was used for 18S rRNA, 5′- CGCAGCTAGGAATAATGGAATAGG-3′ (forward), 5′ -CATGGCCTCAGTT CCGAAA-3′ (reverse), and Platinum SYBR Green qPCR SuperMix-UDG (Invitrogen, Darmstadt, Germany) for GAPDH, 5′-TGGTATCGTGGAAGGAC TCATG-3′ (forward), and 5′-TCTTCTGGGTGGCAGTGATG-3′ (reverse). mRNA expression was determined by normalizing the expression levels separately to the endogenous controls (18S rRNA, GAPDH, and HPRT1) and subsequently calculating the ratio mean values in relation to the gene expression level of the untreated, unimpacted control.

### 4.6. Culture Media Analysis by Means of Commercial ELISA Kits

Biomarker release into culture media (7 days after trauma) was evaluated by means of enzyme-linked immunosorbent assays (ELISAs): secreted MMP-13 was determined using the Human Quantikine ELISA kit (Ray-Biotech, Norcross, GA, USA). Evaluation of type II collagen synthesis was performed using a CPII ELISA (Ibex, Québec, QC, Canada). The assay quantified type II collagen carboxy propeptide (CP II) cleaved from pro-collagen II after its release into the matrix and directly correlated with newly synthetized type II collagen. Degradation of type II collagen was measured using a C2C ELISA (Ibex), detecting a neoepitope generated during collagenase-mediated breakdown of type II collagen. Aggrecanase activity was measured using the Sensitive Aggrecanase Activity Assay from Biotez (Berlin, Germany) for serum-free cell culture supernatants. The kit consists of two modules. First, the substrate (modified aggrecan interglobular domain) is proteolytically cleaved by sample-derived aggrecanases, releasing a peptide with a N-terminal ARGSVIL sequence. This peptide can then be quantified via the specific ELISA module. In principle, the assay quantifies the total aggrecanase activity, comprising that of ADAMTS-1, -4, and -5. 

The total amounts of MMP-13, C2C, and CP II, as well as the aggrecanase activity, were relativized on the weight multiplied by cell viability of the corresponding cartilage explant [41,42].

### 4.7. Gelatin Zymography

Quantification of pro-MMP-2 and active MMP-2 was performed by gelatin zymography, as previously described [41]. In short, culture media (7 days after trauma) were mixed 1:2 with nonreducing zymogram sample buffer (Bio-Rad, Munich, Germany), loaded onto 10% polyacrylamide gels (Carl Roth, Karlsruhe, Germany) containing 2 mg/mL gelatin (Merck, Darmstadt, Deutschland). After electrophoresis (Mini-PROTEAN Tetra Cell System, Bio-Rad), the gels were washed in zymogram renaturation buffer twice for 15 min and incubated in zymogram development buffer for 20 h at 37 °C (both Bio-Rad). Staining with Coomassie solution and subsequent destaining revealed clear bands originating from MMP activity. Band intensities (INT*mm^2^) were quantified with the Geldoc XR system (Bio-Rad) and relativized on tissue weight and cell viability, as mentioned above. An internal standard (positive control) was run on each gel and used to reduce the inter assay variance.

### 4.8. Safranin-O Staining

To evaluate the content of PG within the cartilage tissue, appropriate histological analysis was performed exemplarily (*n* = 1) 14 days after trauma. In short, explants were fixed (4% paraformaldehyde) and embedded in paraffin. Dewaxed and rehydrated sections (3.5 µm) were stained with SafO (Thermo Fisher Scientific, Schwerte, Germany) and Fast Green (Sigma-Aldrich), followed by a final staining of the cell nuclei by Gill’s hematoxylin No. 3 (Sigma-Aldrich) and documentation with an Axioskop 2 mot plus (Zeiss, Oberkochen, Germany).

### 4.9. Statistical Analysis

Experiments were analyzed using GraphPad Prism8 (GraphPad Software Inc., La Jolla, CA, USA). Each data point represented an individual donor (biological replicate); technical replicates from the same donor were not performed. Data sets with *n* ≥ 5 were tested for outliers by means of the Grubbs outlier test. Outliers were not included in statistical analyses. For parametric data sets, a one-way analysis of variance (ANOVA) with the Sidak post-test was used. Nonparametric data sets were analyzed by means of a Kruskal–Wallis test with Dunn’s post-test. For data sets derived from highly degenerated tissue (ICRS grade ≥ 3), an unpaired two-tailed t-test was performed. The significant level was set to a = 0.05. Values in diagrams are given as boxplots (median; whiskers: min to max).

## 5. Conclusions

In conclusion, our results demonstrate the overall importance of the HBP in cartilage homeostasis and degeneration processes, which could be significantly influenced by targeted modification. In this context, we observed predominately chondroprotective and chondroanabolic effects associated with O-GlcNAcylation and the HBP after ex vivo cartilage trauma, implying that alteration of the glycosylation profile might have a detrimental impact on cartilage homeostasis. However, it cannot be excluded that excessive accumulation of O-GlcNAc modified proteins in age might negatively affect cartilage homeostasis in the same manner as its decline, as confirmed by the alteration in the gene expression of HBP-related enzymes in ex vivo traumatized macroscopically intact and in vivo highly degenerated OA cartilage. So far, it is not known whether this reflects a primary pathogenic event or an insufficient protective mechanism in aging and OA, respectively. With respect to various studies reporting contradictory effects of enhanced O-GlcNAcylation in aging, we conclude that this post-transcriptional modification might be a two-edged sword, in particular regarding degenerative diseases. However, in the early post-traumatic situation, the HBP may represent a promising therapeutic target. 

## Data Availability

Data are contained within the article.
